# Computing the biomass potentials for maize and two alternative energy crops, triticale and cup plant (*Silphium perfoliatum* L.), with the crop model BioSTAR in the region of Hannover (Germany)

**DOI:** 10.1186/s12302-014-0019-0

**Published:** 2014-08-10

**Authors:** Roland Bauböck, Marianne Karpenstein-Machan, Martin Kappas

**Affiliations:** 1 Department of Cartography, GIS and Remote Sensing, Research Project ‘BIS’, University of Göttingen, Goldschmidtstraße 5, Göttingen, 37077 Germany; 2 Interdisciplinary Centre of Sustainable Development, Research Project ‘BIS’, University of Göttingen, Goldschmidtstraße 1, Göttingen, 37077 Germany

**Keywords:** Alternative energy crops, BioSTAR, Biomass potentials, Integrative concepts

## Abstract

**Background:**

Lower Saxony (Germany) has the highest installed electric capacity from biogas in Germany. Most of this electricity is generated with maize. Reasons for this are the high yields and the economic incentive. In parts of Lower Saxony, an expansion of maize cultivation has led to ecological problems and a negative image of bioenergy as such. Winter triticale and cup plant have both shown their suitability as alternative energy crops for biogas production and could help to reduce maize cultivation.

**Results:**

The model Biomass Simulation Tool for Agricultural Resources (BioSTAR) has been validated with observed yield data from the region of Hannover for the cultures maize and winter wheat. Predicted yields for the cultures show satisfactory error values of 9.36% (maize) and 11.5% (winter wheat). Correlations with observed data are significant (*P* < 0.01) with *R* = 0.75 for maize and 0.6 for winter wheat. Biomass potential calculations for triticale and cup plant have shown both crops to be high yielding and a promising alternative to maize in the region of Hanover and other places in Lower Saxony.

**Conclusions:**

The model BioSTAR simulated yields for maize and winter wheat in the region of Hannover at a good overall level of accuracy (combined error 10.4%). Due to input data aggregation, individual years show high errors though (up to 30%). Nevertheless, the BioSTAR crop model has proven to be a functioning tool for the prediction of agricultural biomass potentials under varying environmental and crop management frame conditions.

## Background

### Current state biogas production

Bioenergy from agricultural substrates (plant material and manure) has become an important input to the renewable energy mix in Germany [1] particularly in Lower Saxony [2]. Lower Saxony is the most important agricultural state in Germany [3], with the highest installed electric capacity from biogas [4]. The dominant plant substrate used in these facilities is maize grown for silage [5]. Silage maize is well established in Germany as a feed culture in cattle farms. Through years of successful breeding, the thermophile C_4_ crop has been adapted to the central European climate, and with ample water and temperature, high biomass yields are possible. This aspect makes maize the most widely used energy crop for biogas production in Lower Saxony and Germany in general. The marginal returns are high; especially farmers in regions with a high cattle stocking are familiar with this crop, and risks associated with the production of silage maize are low and known to the farmers. With a boom of bioenergy facilities stimulated by the German renewable energies act (first version 2000), a strong expansion of maize production in Germany and in Lower Saxony has been triggered [6]. From 2001 to 2012, the number of biogas-producing facilities has increased from 148 to 1,480 [7]. As a result of this strong expansion of the maize share in the crop mix on agricultural land (some of the expansion was generated by turning pasture into cropland or by using low-yielding lands which had been used as fallow and thus to some degree for nature conservation), criticism against a further expansion of bioenergy crop production and against existing biogas (methane) facilities arose. The main points of concern with regard to the strong expansion of the maize production are as follows [8]:

1. A reduction of biodiversity (in species related to crops and crop rotations)
2. A negative impairment of the characteristic landscape (dominated by cereals for centuries)
3. Ecologically adverse effects like nitrate leaching, soil erosion and a reduction of humus content in the soils

According to the German environmental protection law, farmers need to follow certain agricultural guidelines referred to as ‘good professional practice’ [9]. According to paragraph 1 of the German environmental protection law, the diversity, character and beauty of the landscape have to be protected; their state is not to be worsened and has to be restored where damage or impairment has occurred. According to paragraph 5 of this law and according to the guidelines of a good professional practice, the long-term maintenance of soil fertility and the practice of maintaining a crop rotation with three different crops have to be established. Furthermore, it is stipulated that the natural fitting of soil, water, flora and fauna should not be impaired beyond the measure of a renewable yield of an agricultural site [10].

Nevertheless, large-scale cultivation of maize for silage (as is the case in some parts of Lower Saxony) leads to monocultures and can cause the environmental problems mentioned above.

One possibility to counteract the negative impacts associated with the large-scale production of silage maize would be the introduction of new bioenergy crops into the existing crop rotations and, beyond this, to create new ‘integrative concepts’ [11,12].

### Integrative concepts with new energy crops

The term ‘integrative concept’ is used for the scientific approach which combines different land use options to produce food, fodder and energy while promoting biodiversity and ecosystem services in agricultural landscapes [13-16]. Integrative cultivation concepts should harmonize utilization and protection of a landscape. Due to external costs of non-sustainable systems, only sustainable concepts are economically sound in the long run. The vision of integrative concepts is to contribute to a more diverse agricultural landscape, keep nature in balance and conserve ecosystems. The integration of new annual energy crops into the crop rotation, as well as the cultivation of perennial crops on problematic soils, offers great opportunities to mitigate negative impacts of agriculture on biodiversity and ecosystem services. In this article, we will focus on two new energy crops (triticale and *Silphium perfoliatum* L.) which have the potential to increase crop and landscape diversity.

#### Triticosecale wittmack *(triticale)*

As an energy crop, winter triticale is suitable for locations with cool and moderate climates and locations that lack a high summer precipitation. Triticale utilizes winter soil moisture to produce biomass in the spring. It already reaches the maximum biomass yield in the first half of the year. Therefore, it is hardly affected by summer dryness. Dry matter yields range between 12 and 16 t/ha. Triticale is grown on good soil quality locations in southern Lower Saxony, and it is the most productive winter crop for biogas production. Even on poorer soils, triticale produces high biomass yields.

In combination with field grass (double cropping system), triticale can broaden a narrow maize rotation, increase biodiversity and improve the humus balance [17].

#### Silphium perfoliatum *L.*

A very long useful life is anticipated for Silphie (*S. perfoliatum* L.), also known as ‘cup plant’. With its cupped leaves, Silphie can collect air moisture; it is therefore relatively resistant to dry conditions. It is adapted to the moderate climate conditions of eastern North America and can be cultivated 400 m above sea level. Silphie has been cultivated as fodder for cattle in North America and in the former German Democratic Republic (GDR). It was tested as an alternative biogas crop in field trials in Germany from 2005 onward. In 2010, farmers cultivated Silphie on about 20 ha of farmland [18]. The best results have been obtained when the seeds are sown and nursed in greenhouses and transplanted as young plants with three or four leaves into the fields in May or June. In the first year, the crop should establish itself in the soil and the plants should only build a leaf rosette before winter. In the following spring, the plants grow very quickly and can deliver their first harvest in early autumn [19]. The first results show that Silphie has a very high yield which is similar to that of maize [20,21]. One big advantage of this crop is the fact that after the first year, it needs no further weed control and no additional pesticides. Further advantages are given by the absence of a yearly tillage, which induces CO_2_ storage in the soil and by the long flowering period of Silphie which is used by honeybees to collect pollen and nectar (bee bread) to survive and reproduce. However, the seed quality of this crop still must be improved to help broaden Silphie's use as a commonly used energy crop.

## Results and discussion

### Model validation

To verify the biomass results of the model Biomass Simulation Tool for Agricultural Resources (BioSTAR) [22], winter wheat and maize yields for the years 1981 to 2007 for the region of Hannover have been calculated and then compared with observed yield data from the same years and region and then statistically analysed.

In Table 1, the observed (obs.), the predicted (pred.), the percent error of the predicted (e%), the adjusted value of the prediction (adjust) and the percent error of the adjusted value of prediction (ae%) are displayed for both maize and winter wheat for the years 1981 to 2007. Below the individual year listings, mean values for each column, the adjustment factor, the root-mean-square error (RMSE), the overall percent error (calculated from the RMSE) and the *Willmott index of agreement* are given for both crops.

**Table 1:** **Comparison of observed (statistical data) and predicted (modelled) yields for maize and winter wheat, 1981–2007**

**Year**	**Maize**					**Winter wheat**				
	**obs.**	**pred.**	**e%**	**adjust**	**ae%**	**obs.**	**pred.**	**e%**	**adjust**	**ae%**
1981	482.9	753.1	55.9	542.2	12.3	55.1	90.7	64.7	72.6	31.7
1982	491.6	650.7	32.4	468.5	−4.7	64.5	78.7	22.0	62.9	−2.4
1983	400.4	533.2	33.2	383.9	−4.1	61.3	85.8	39.9	68.6	11.9
1984	413.2	642.9	55.6	462.9	12.0	63.3	96.5	52.5	77.2	22.0
1985	468.3	794.2	69.6	571.8	22.1	61.8	87.5	41.5	70.0	13.2
1986	460.5	715.7	55.4	515.3	11.9	78.3	82.0	4.7	65.6	−16.3
1987	427.6	703.0	64.4	506.2	18.4	75.4	83.5	10.8	66.8	−11.3
1988	458.4	528.8	15.4	380.8	−16.9	71.5	73.8	3.2	59.0	−17.4
1989	414.2	418.0	0.9	301.0	−27.3	54.0	79.7	47.7	63.8	18.1
1990	379.8	516.2	35.9	371.6	−2.2	71.9	79.0	9.9	63.2	−12.1
1991	399.0	492.9	23.5	354.9	−11.1	81.3	93.8	15.4	75.0	−7.7
1992	367.8	382.0	3.9	275.1	−25.2	75.3	78.0	3.6	62.4	−17.1
1993	461.0	704.5	52.8	507.2	10.0	82.9	97.5	17.6	78.0	−5.9
1994	429.1	638.6	48.8	459.8	7.2	81.5	96.1	17.9	76.9	−5.6
1995	398.3	569.9	43.1	410.3	3.0	84.2	102.56	21.8	82.1	−2.6
1996	424.7	639.3	50.5	460.3	8.4	82.4	83.4	1.3	66.7	−19.0
1997	460.9	695.1	50.8	500.4	8.6	88.5	111.25	25.7	89.0	0.6
1998	454.5	655.9	44.3	472.3	3.9	82.0	102.68	25.3	82.2	0.2
1999	432.6	632.0	46.1	455.0	5.2	93.5	102.42	9.5	81.9	−12.4
2000	496.3	582.3	17.3	419.3	−15.5	87.7	98.5	12.2	84.8	−3.4
2001	489.7	619.3	26.5	445.9	−8.9	95.4	107.67	12.8	91.8	−3.8
2002	470.7	650.1	38.1	468.1	−0.6	76.4	117.61	54.0	99.3	30.0
2003	375.2	441.1	17.6	317.6	−15.3	78.8	88.2	11.9	73,44	−6.8
2004	480.6	722.4	50.3	520.2	8.2	89.9	125.68	39.7	103.8	15.4
2005	482.2	711.5	47.6	512.3	6.2	84.8	121.74	43.6	99.5	17.3
2006	421.5	581.0	37.9	418.3	−0.7	84.3	102.66	21.8	93.0	−1.6
2007	537.7	771.4	43.5	555.4	3.3	77.5	107.48	38.7	86.0	10.9
	Mean	Mean	Mean	Mean	Mean	Mean	Mean	Mean	Mean	Mean
	445.1	624.3	39.8	449.5	0.63	77.2	95.4	24.8	77.3	1.0
	Factor adjust		0.72			Factor adjust		0.80		
	RMSE (dt/ha)		42.9			RMSE (dt/ha)		9.6		
	% error		9.6			% error		11.5		
	WIA		0.89			WIA		0.74		

Data sources: BioSTAR data and LSN. obs., observed yield; pred., predicted yield; e%, percent error of the predicted; adjust, adjusted value of the predicted; ae%, percent error of the adjusted value of prediction. Values are in deci-tons per hectare (dt/ha) fresh mass for maize and dt/ha grain weight for winter wheat. Mean, mean value of column; RMSE, root-mean-square error; WIA, Willmott index of agreement.

The adjusted yield value is a product of the predicted yield value and the adjustment factor (factor adjust).

The adjustment factor is needed here to adjust the output of the model (which has been calibrated with field trial data) to realistically achievable yields of actual agriculture. The yields of actual agriculture can be up to 30% lower than those achieved in controlled field trials. Possible explanations for this are more homogenous soils and optimal crop care (pest and weed control and fertilizer application) on small trail parcels of several square metres compared to large agricultural lots of many hectares.

To derive this adjustment factor, the mean of the observed yield has been divided by the mean of the predicted yield. For maize, the resulting adjustment factor is 0.72 (72%), and for winter wheat, it is 0.80 (80%). Four years, 1989 and 1992 for maize and 1981 and 2002 for winter wheat, show higher deviations of the predicted from the observed yield values.

For winter wheat, the yield was overestimated by 31.7% in 1981 (after the adjustment) and 64.7% (before the adjustment). A possible explanation for this is an unusually wet spring/early summer with rainfall amounts of 108 and 146 mm in May and June. Compared to the long-term (1981 to 2010) averages for these months in the region of Hannover (56 and 65 mm), this is a surplus of 93% and 125% of rainfall. The same holds true for the year 2002 (overestimation of 30%) where July was extremely wet with average rainfall amounts of 171 mm. This is a surplus of 160% in comparison to the average (67 mm) from the period 1981 to 2010 for this region and month.

The model uses these additional litres of water to generate an increased crop growth. Possibly adverse effects due to continuous rain, like water-logged soils (not modelled in this case due to limited soil data), or humidity-related fungus problems are not accounted for. Strongest deviations for maize show up for the years 1989 and 1992, where yields have been underestimated by 27.3% and 25.2% after the adjustment factor (0.72) has been applied to the simulation data.

Since winter wheat is not grown on low-quality soils, only model results from soil data with *nFK* values >90 mm have been used for the comparison of the observed and predicted winter wheat yields. For maize, which is usually grown on a big span of soil types, only very poor quality soils with field capacity values below 40 mm have been omitted from the comparison.

**Figure 1:**
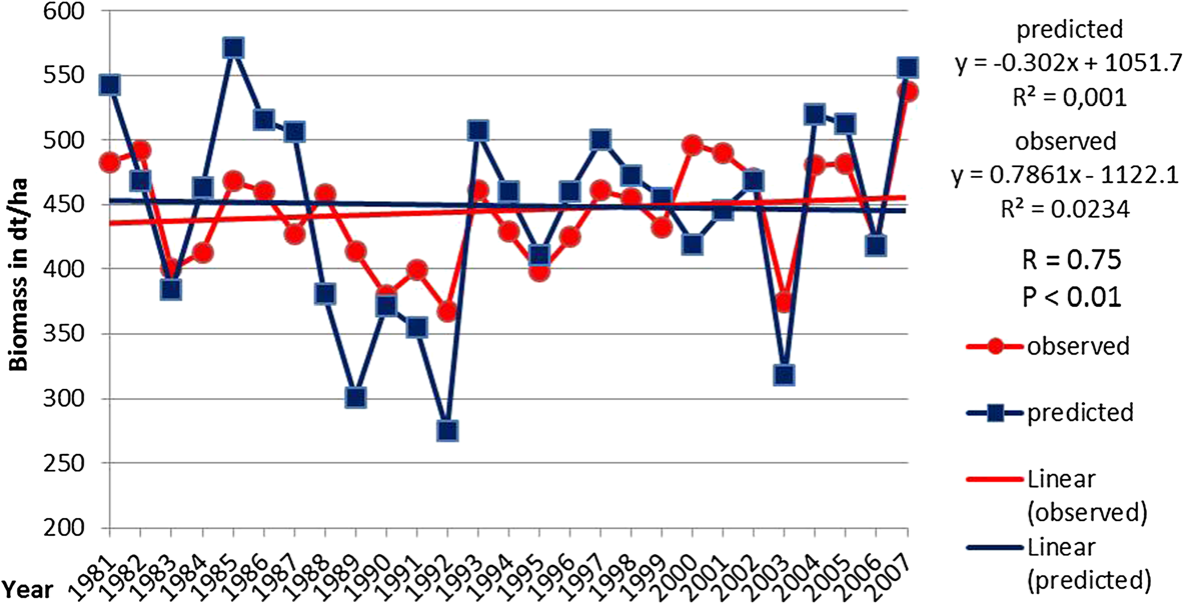
**Observed and predicted maize yields on the timeline (1981 to 2007) and linear regression analysis for both.**

**Figure 2:**
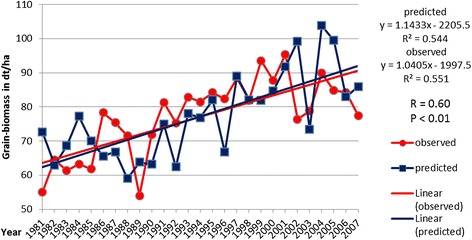
**Observed and predicted winter wheat yields on the timeline (1981 to 2007) and linear regression analysis for both.**

In Figures 1 and 2, yield trends (observed and predicted) across the analysed 27 years for maize and winter wheat and their linear regressions are displayed. The regression lines for maize more or less show stagnating yields over the years, and the regressions for winter wheat show a trend of increasing yields in the time slot. For the observed winter wheat data, the explanation lies in advances in breeding. For the simulated yields, the advances in breeding have been accounted for after the simulation run. Deductions for all years before 2000 following the regression line of the observed yields have been made. This is necessary because the model is calibrated to fit with yield data from the period 2005 to 2010 (current state breeding). After 2000, no statistically significant yield gains through breeding can be accounted for. For both maize and winter wheat, the correlation (observed vs. predicted values) is significant with *P* < 0.01 and *R* = 0.75 for maize and *R* = 0.60 for winter wheat. The overall fit of the predicted yield data (after the adjustment) with the observed data for all years combined is at a good level for both cultures with satisfactory statistical error values. The RMSE values for maize and winter wheat are at 42.9 dt/ha (deci-tons per hectare) fresh mass (maize) and 9.6 dt/ha grain weight (winter wheat); the percent errors are at 9.36% (maize) and 11.5% (winter wheat), and the Willmott index of agreement is at 0.89 for maize and 0.74 for winter wheat.

### Biomass potentials in the region of Hannover

Biomass potentials (dry matter yields per hectare) for maize, triticale and cup plant have been calculated with the crop model BioSTAR. In a second step, these yields have been joined with a geographical information system (GIS) map of the soil dataset (*Bodenschätzungsdaten*/soil evaluation data) of the region of Hannover [23]. This map has a total of 114,357 individual soil units, divided into eight different soil types and five different climate regions (see ‘Methods’ section). The yield data has been joined with the procedure described in the ‘Methods’ section. Because the years 1990 to 2007 are more representative for the current state climate (dryer summers, higher temperatures), only these years have been used for the biomass potential calculation of the three cultures.

**Figure 3:**
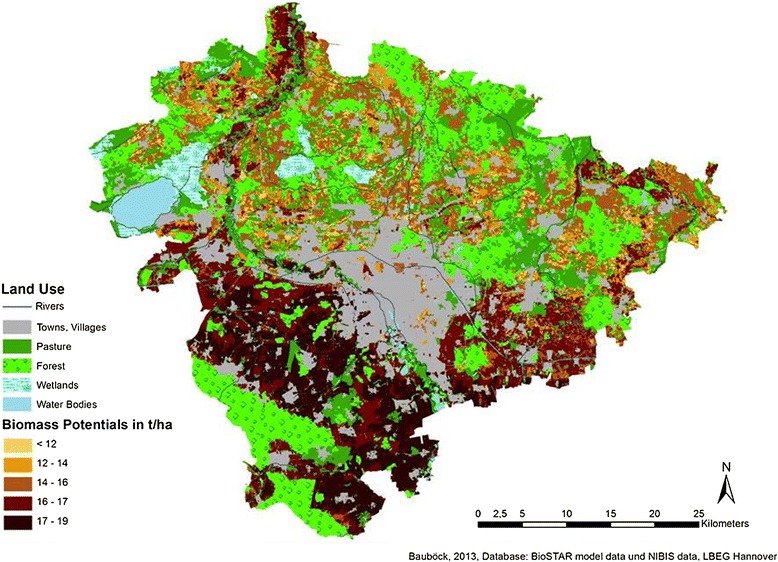
**Biomass potentials for maize in the region of Hannover (climatic period 1991 to 2007).**

**Figure 4:**
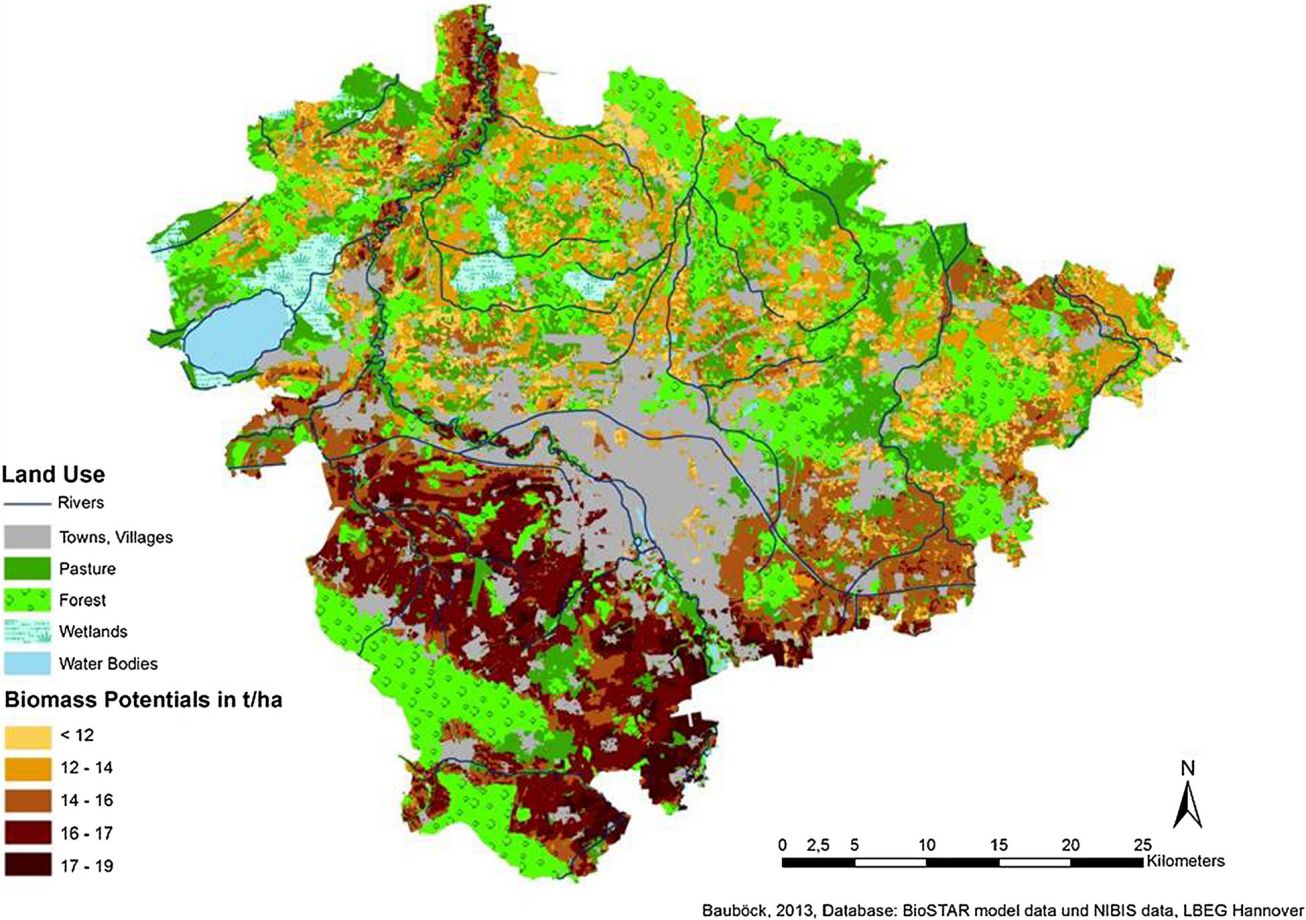
**Biomass potentials for cup plant (** ***Silphium perfoliatum*** **) in the region of Hannover (climatic period 1991 to 2007).**

**Figure 5:**
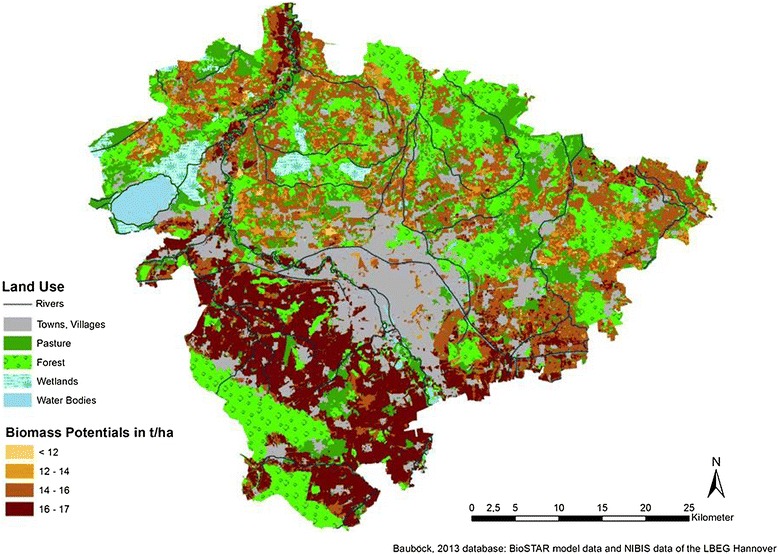
**Biomass potentials for triticale in the region of Hannover (climatic period 1991 to 2007).**

**Figure 6:**
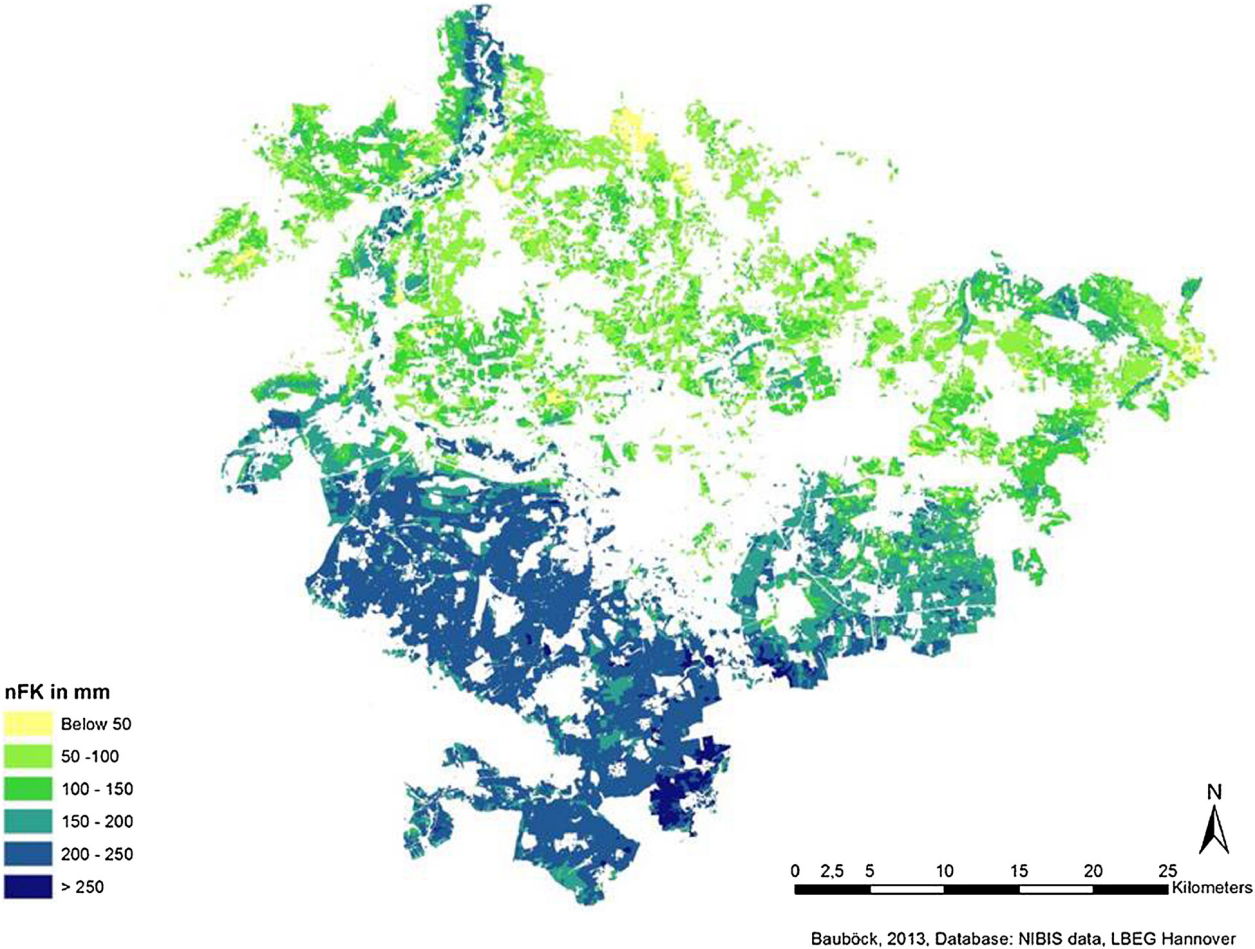
**Field capacity in the rooted zone in the region of Hannover in millimetres.**

In Figures 3, 4, and 5, the results of the joined data are cartographically displayed. Figure 6 shows the spatial distribution of the *nFK* values in the region. For triticale and cup plant, no adjustment factor (yields from field trials vs. actual yields) could be determined due to lack of statistical data. Hence, for triticale and for cup plant, the same factor was used as for winter wheat (0.8). The distribution of the biomass potentials for all three cultures shows a similar pattern roughly following the distribution of the *nFK* values (and thus the soil type distribution) in the region. All three cultures profit from the occurrence of the loamy and silty type soils and the higher *nFK* values (150 to 260 mm) in the southern part of the region and produce the highest yields there. The northern part of the region is mostly dominated by sandy type soils (*nFK* values below 150 mm), with an exception of the ‘Leine’ river valley in the northwest. Maize, a C_4_-culture with a high yield potential, does show up with the highest biomass potentials of the three crops. In the southern part of the region, maize has average potentials between 16 and 19 t dry mass/ha. In the northern part (except for the Leine valley), the potentials are lower and range between 12 and 16 t/ha. Soils with potentials below 12 t/ha are only few.

For cup plant, the biomass potentials are not as high as for maize. However, yields between 16 and 17 t/ha are frequent in the south, and on some soils, yields of up to 19 t/ha can be reached. The northern part, dominated by the sandy type soils, has average biomass potentials around 12 to 14 t/ha (except for the Leine valley, where it is higher), but on some soils, the potentials can be lower than 12 t/ha.

Triticale shows a different biomass potential distribution pattern than the other two cultures. The division into north and south can still be recognized here, but the differences are less pronounced.

The high yield stability of triticale on a wide span of soil qualities has been found in previous research as well (see [16]). Triticale has average potentials between 16 and 17 t/ha in most parts of the southern region and yield potentials between 14 and 16 t/ha in the north. Soils with yield potentials below 12 t/ha and even below 14 t/ha are few.

As an overall conclusion for the three cultures, it can be said that all of them are interesting crops for biomass production in the region due to their overall high biomass potentials. For the high-quality soils in the southern part of the region, all three cultures have high potentials of 16 t/ha or more. In the north, cup plant appears to have the lowest potentials and maize and triticale are very similar.

## Conclusions

The calculation of the spatial distribution of the biomass potentials for two alternative energy crops (triticale and cup plant) and maize in the region of Hannover has been broken down into two steps. In the first step, the crop model BioSTAR (see [22]) was used to calculate the biomass potentials for maize and winter wheat with climate data (period 1981 to 2007 and five different climate stations) and soil data (35 soil and *nFK* classes). The generated biomass yields have then been compared with statistical yield data (obtained from the LSN), and a correction factor was deduced from this comparison. In the second step, the biomass yields for triticale and cup plant have been calculated with the model, and the generated yields for maize, triticale and cup plant (after correction) have been connected to a GIS-soil map of the region to visualize the spatial distribution of the biomass potentials for the three cultures.

The first step of the modelling has shown that BioSTAR was able to predict the aggregated yearly yields for winter wheat and maize in the region at a satisfactory level of accuracy, but individual years stand out with stronger than average (9.6% for maize and 11.2% for winter wheat) deviation of up to 31.7% from the observed yield. Possible explanations for these model deviations lie in the resolution of the input data. Both the climate and the soil data used for the simulations are strongly aggregated datasets. The climate data is a monthly aggregation from five representative climate stations in the region, and the soil data has been aggregated into 35 soil types and *nFK* classes. Additionally, only homogenous soil profiles have been assumed as inputs for the model. All these aggregations make it possible to predict biomass potentials and their spatial distribution for a large region, like the region of Hannover, with a reasonable amount of time and work input. Previous BioSTAR validation procedures with non-aggregated climate and soil data have produced better fits of observed and predicted yield data [24]. The biomass potential calculation presented in this article serves as a demonstration of the method itself and to give an overview and comparison of the relative biomass potentials of maize and the two modelled alternative energy crops, triticale and cup plant. Depending on the exactness and resolution of the input data, a higher degree of model prediction accuracy can be achieved.

The method (biomass calculation with the crop model BioSTAR) offers itself as a tool for a wide span of applications concerning a sustainable biomass production and production allocation on agricultural sites.

Based upon existing bioenergy facilities in a landscape and their respective claim of acreage, expansion scenarios can be defined and potential areas for biomass usage can be located.

This has been done in a series of workshops in the region of Hannover. In the first step, bioenergy expansion scenarios were defined in cooperation with the local government and representatives from agriculture institutions and farmers. Based upon sustainability criteria and taking mixed crop rotations with food crops, feed stuff and alternative energy crops into account, existing bioenergy potentials have been identified.

Protected (nature conservation) areas were omitted in this analysis. Through GIS visualization, suitable areas for bioenergy production could be found. Additional criteria like the prevalence of heat sinks, farms with animal manure production or other residual materials could be accounted for in this step.

Additionally, the method offers applications which go beyond the use of biomass in a region. Other potential applications are for instance to show how different crop rotations and possible changes in climate will affect the agricultural potential in a region or on a farm. Therefore, the method appears to be a useful tool for the prediction of agricultural potentials under varying environmental and crop management frame conditions.

## Methods

### Model and model verification

Biomass calculations for maize, winter wheat, winter triticale and cup plant (*S. perfoliatum*) have been performed with the crop model BioSTAR (see [22]). BioSTAR is a new crop model which was originally developed for the prediction of site-specific biomass potentials for bioenergy crops, mainly maize and cereals for silage. The model is capable of modelling the biomass and yield potentials for a number of agricultural crops, including perennial crops like cup plant (*S. perfoliatum* and short rotation coppices). Due to the software architecture of the model (MS Access® database interface), large numbers of individual sites can be modelled in one single procedure and data editing for large data files, containing soil and climate datasets of whole regions, is easily manageable.

BioSTAR offers four different growth calculation methods (growth engines) and four different ET_0_ calculation methods. Because most of the model testing and calibration performed up to now were done with the CO_2_-based growth engine and the photosynthesis rate-dependent transpiration method, these two have been used for the calculation of the biomass potentials described in this article.

As a cross-check and to verify the model generated data, the results of the maize model run and the results of an additional model run with the culture winter wheat have been compared to actual harvest data from harvest statistics of the region of Hannover from the years 1981 to 2007 [25]. The harvest data values are arithmetic means of all the reported yields of an individual year and can be interpreted as representative of a whole region, in this case the region of Hannover.

For this comparison, the biomass yields of maize (model output is in tons dry mass per hectare) had to be converted to deci-tons (dt) fresh mass (unit of the harvest statistic). Because the dry matter content of the yields is probably rarely recorded and, if so, it definitely does not find its way into the highly aggregated statistical data, a value of 35% dry matter content for maize harvested for silage has been assumed.

The winter wheat yields in the statistics are given in deci-tons grain yield (per hectare). To convert the model output which is given in tons dry matter per hectare (DM [t/ha]) into these units, a linear regression (Equation 1) from Moeser [26] describing the relation between biomass yield (DM) and grain yield (Grain) in winter cereals has been used.

1 $$ \mathrm{Grain}\kern0.37em \left[\mathrm{dt}/\mathrm{ha}\right]=\left(\mathrm{DM}\ \left[\mathrm{t}/\mathrm{ha}\right]\times 10+51.377\right)/2.5188 $$

### Processing of soil and climate data

The basis for the input data used with the BioSTAR model is a soil dataset (*Bodenschätzungsdaten*/soil evaluation data) of the region of Hannover with a very high spatial resolution (1:5,000). The relevant information contained in the soil dataset is the dominant soil texture class (soil type) at the individual sites and the German soil classification index number, the *Bodenzahl* (in the following referred to as the *soil number*). The soil number is a quality measure for agricultural soils ranging from below 20 (poor-quality soil) to 100 (highest quality soil).

Following Müller et al. [27], a calculation procedure has been applied to calculate the plant available field capacity (in the following referred to as *nFK*) for each individual soil evaluation site in the region of Hannover. With the knowledge of the soil number and the soil type of a site, the field capacity value can be taken from a reference table. The tabulated *nFK* values (Table 2) were then used to generate linear and polynomial fit equations to approximate *nFK* values for any given soil number and soil type. In total, there are eight soil types and seven *nFK* classes.

**Table 2:** **Reference table for approximation of** ***nFK*** **values from soil type and soil number**

	***nFK*** **classes [mm] and the corresponding class boundaries of the soil numbers**						
	**50**	**90**	**140**	**200**	**250**	**270**	**300**
S	≤21	>21 ≤ 37	>37				
Sl	≤17	>17 ≤ 31	>31 ≤ 47	>47			
lS	≤18	>18 ≤ 32	>32 ≤ 49	>49 ≤ 71	>71		
SL	≤17	>17 ≤ 31	>31 ≤ 48	>48 ≤ 69	>69 ≤ 86	>86	
sL	≤18	>18 ≤ 33	>33 ≤ 51	>51 ≤ 73	>73 ≤ 92	<92	
L	≤17	>17 ≤ 31	>31 ≤ 48	>48 ≤ 69	>69 ≤ 86	>86 ≤ 95	>95
LC	≤21	>21 ≤ 38	>38 ≤ 59	>59 ≤ 84	>84		
C	≤21	>21 ≤ 38	>38 ≤ 60	>60			

Source: Changed, after Müller et al. [27]. S, sand; Sl, slightly loamy sand; lS, loamy sand; SL, very loamy sand; sL, sandy loam; L, loam; LC, heavy loam; C, clay.

**Figure 7:**
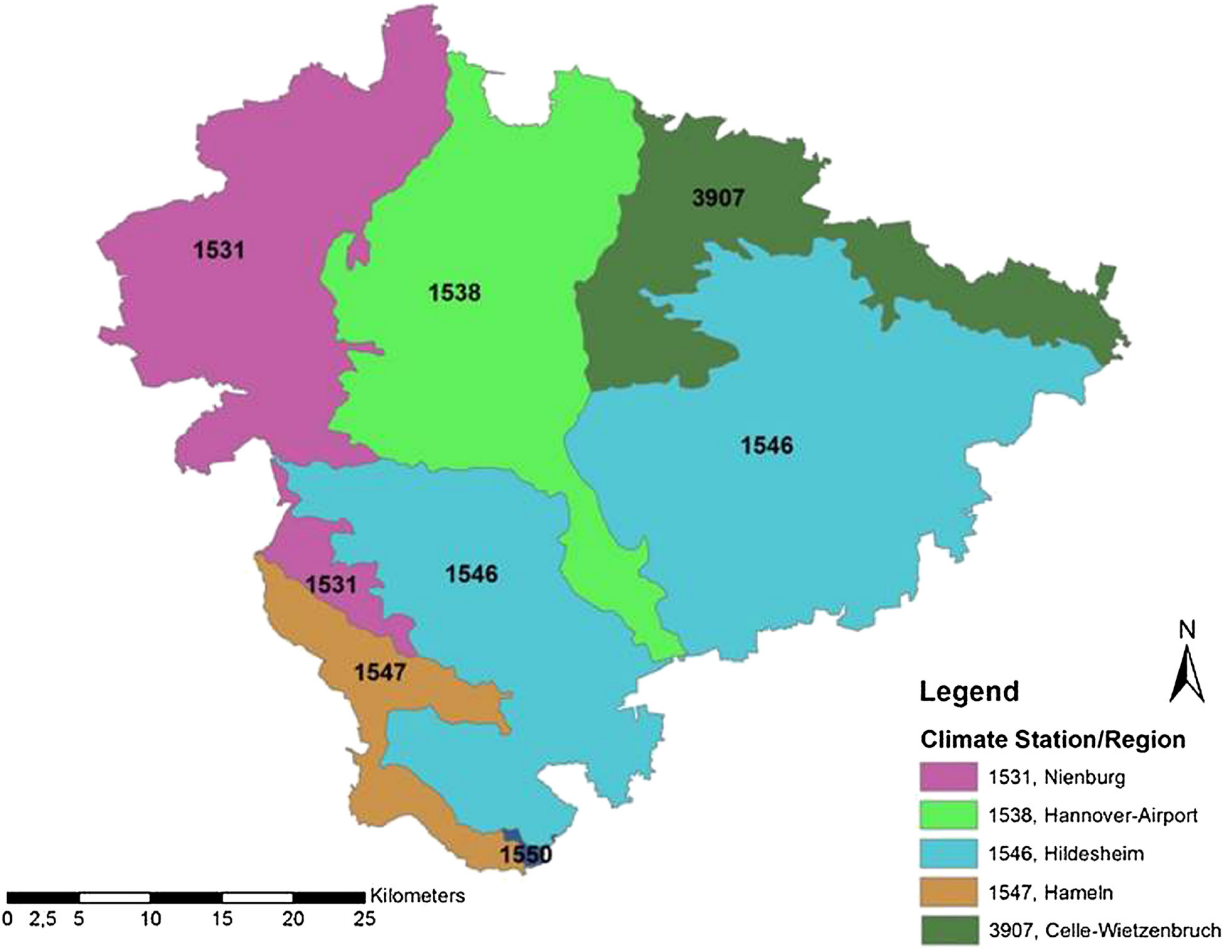
**Discrete climate regions with DWD station number in the region of Hannover.**

The soil evaluation map of the region of Hannover contains more than 114,000 sites. To shorten the calculation procedure of the BioSTAR model and to avoid redundancy, only the biomass yields of three to five *nFK* classes (depending on the *nFK* span of the soil type) for each soil type have been calculated with the model. Additionally, the calculation was differentiated by using climate data from five different DWD climate stations in the region of Hannover (Figure 7), each representing a homogenous climate region (on the basis of long-range climate measurements) (data taken from NIBIS®). From each climate station, the daily climate values from 1981 through 2007 have been converted into monthly mean values. On the basis of these monthly mean values of the five stations, the BioSTAR calculations have been performed. As a result, a very large number of sites (>114,000) with 8 dominant soil types and a climatic variability expressed in the data of 5 climate stations (Figure 7) was converted into 175 different data units (35 soil and *nFK* classes × 5 climate zones) to be processed for each individual year (1981 to 2007). Using this method allows the aggregation of the >114,000 soil sites into 175 data units and thus eliminates redundancies and shortens the calculation procedure.

The resulting biomass yields (175 data units × 27 years) for the three modelled crops (maize, triticale, cup plant) have then been averaged over the whole time period (1981 to 2007).

**Figure 8:**
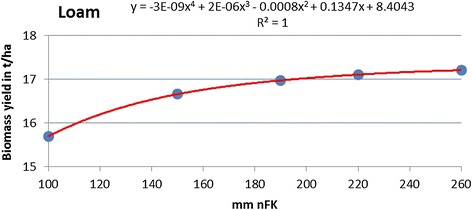
**Polynomial fit for** ***nFK*** **-dependent yield (triticale on loam).**

**Figure 9:**
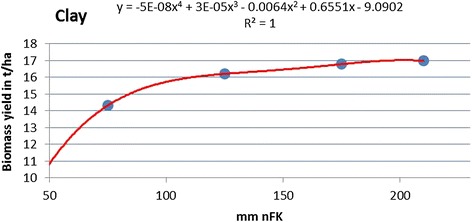
**Polynomial fit for** ***nFK*** **-dependent yield (triticale on clay).**

For the transfer of the aggregated yield data (35 *nFK* classes, 5 climate regions) to the actual GIS (geographical information system) soil type data table, polynomial equations describing the relation between the *nFK* class of each soil type and the corresponding yield for each crop have been generated. These polynomial fit curves thus serve as data interpolation curves and are not meant to describe the accuracy of the fit, and only *nFK* values within an upper and a lower threshold have been used (thus no *x* values below zero can occur). As an example, the polynomial fit curves for triticale on loam and clay are displayed (Figures 8 and 9).

Loamy soils (these include silts in this classification system), which are generally of a high quality, start at much higher *nFK* values than clays (100 mm vs. <50 mm). Yield gains on loam are strong between 100 and 150 mm of available soil water and then decline. The curve for clay type soils shows a strong increase in productivity for *nFK* values between 50 and 100 mm and then levels of an inclination similar to that of the loam curve.

## Abbreviations

BioSTAR: Biomass Simulation Tool for Agricultural Resources
C_4_ culture: crop with a C_4_ carbon pathway
dt: deci-tons
DWD: Deutscher Wetterdienst
ET_0_: potential (reference) evapotranspiration rate
GDR: German Democratic Republic
GIS: geographical information system
LWK: Landwirtschaftskammer (Niedersachsen)
LSN: Lower Saxony Department of Statistics
*nFK*: available field capacity
NIBIS®: Lower Saxony soil information system
RMSE: root-mean-square error
WIA: Willmott index of agreement
